# Inhibition of p38 MAPK Signaling Augments Skin Tumorigenesis via NOX2 Driven ROS Generation

**DOI:** 10.1371/journal.pone.0097245

**Published:** 2014-05-13

**Authors:** Liang Liu, Hamid Reza Rezvani, Jung Ho Back, Mohsen Hosseini, Xiuwei Tang, Yucui Zhu, Walid Mahfouf, Houssam Raad, Grace Raji, Mohammad Athar, Arianna L. Kim, David R. Bickers

**Affiliations:** 1 Department of Dermatology, Columbia University Medical Center, New York, New York, United States of America; 2 Biothérapies des maladies génétiques et cancers, Univ. de Bordeaux, Bordeaux, France; 3 INSERM, Biothérapies des maladies génétiques et cancers, Bordeaux, France; 4 Department of Dermatology, University of Alabama at Birmingham, Birmingham, Alabama, United States of America; University of Tennessee, United States of America

## Abstract

p38 mitogen-activated protein kinases (MAPKs) respond to a wide range of extracellular stimuli. While the inhibition of p38 signaling is implicated in the impaired capacity to repair ultraviolet (UV)-induced DNA damage—a primary risk factor for human skin cancers—its mechanism of action in skin carcinogenesis remains unclear, as both anti-proliferative and survival functions have been previously described. In this study, we utilized cultured keratinocytes, murine tumorigenesis models, and human cutaneous squamous cell carcinoma (SCC) specimens to assess the effect of p38 in this regard. UV irradiation of normal human keratinocytes increased the expression of all four p38 isoforms (α/β/γ/δ); whereas irradiation of p53-deficient A431 keratinocytes derived from a human SCC selectively decreased p38α, without affecting other isoforms. p38α levels are decreased in the majority of human cutaneous SCCs assessed by tissue microarray, suggesting a tumor-suppressive effect of p38α in SCC pathogenesis. Genetic and pharmacological inhibition of p38α and in A431 cells increased cell proliferation, which was in turn associated with increases in NAPDH oxidase (NOX2) activity as well as intracellular reactive oxygen species (ROS). These changes led to enhanced invasiveness of A431 cells as assessed by the matrigel invasion assay. Chronic treatment of p53^-/-^/SKH-1 mice with the p38 inhibitor SB203580 accelerated UV-induced SCC carcinogenesis and increased the expression of NOX2. NOX2 knockdown suppressed the augmented growth of A431 xenografts treated with SB203580. These findings indicate that in the absence of p53, p38α deficiency drives SCC growth and progression that is associated with enhanced NOX2 expression and ROS formation.

## Introduction

Exposure to solar ultraviolet (UV) radiation is a primary risk factor for human skin carcinogenesis. In addition to its ability to induce mutagenic DNA damage, UV radiation induces extensive cellular damage through a variety of mechanisms, including augmented production of intracellular reactive oxygen species (ROS) [1]. Augmented ROS and consumption of physiological antioxidants drive signaling mechanisms involved in tumor promotion and progression [1]–[3]. Intracellular ROS are primarily generated through aerobic metabolism or through a specialized group of enzymes, known as the NADPH oxidases, in which NOX is the catalytic subunit [4], [5]. NADPH oxidase activity is associated with several characteristic features of cancer, including genomic instability, cell proliferation, survival, invasion, and metastasis [6]–[8]. Of the seven distinct NOX enzymes (NOX1–NOX5, Duox1, and Duox2) that are known to exist in humans, NOX1-induced ROS has been implicated in oncogenic signaling in Ras-transformed NIH3T3 cells [9]. Moreover, increases in NADPH oxidase activity and NOX1 levels are observed in human cutaneous squamous cell carcinomas (SCCs) and keratinocytes of individuals affected with xeroderma pigmentosum C (XPC), an autosomal recessive disorder that is associated with compromised nucleotide excision repair that leads to accelerated development of multiple types of skin cancer [10], [11]. Despite the evidence supporting the role of oxidative stress in skin cancer, the molecular pathways involved in skin carcinogenesis are not fully understood.

p38 mitogen-activated protein kinases (MAPKs) are activated in response to a wide range of extracellular stimuli, including among others osmotic and thermal stress, growth factors, inflammatory cytokines, and UV radiation. p38 influences various cellular processes, including proliferation, differentiation, apoptosis, and inflammation [12]. Four p38 MAPK isoforms have been identified, including α, β, γ, and δ. Among these four isoforms, p38α and p38β have overlapping functions; meanwhile, p38γ and p38δ are structurally similar to each other, but distantly related to both p38α and p38β [13] [14]. Furthermore, p38α and p38δ are abundantly expressed in epidermis, whereas p38β or p38γ are virtually undetectable in normal epidermis [15], [16]. p38α has also been shown to be responsive to UV irradiation and plays an important role in the regulation of cell-cycle arrest and apoptosis [17]. The exact role of p38α in cancer, however, remains controversial. Both anti-proliferative and survival functions of p38α have been described [18]–[21]. For example, p38α can negatively regulate cell cycle progression at both the G1/S and G2/M transitions, via downregulation of cyclins and upregulation of cyclin-dependent kinase inhibitors [18], [19]. In contrast, pro-survival roles of p38 have also been observed during the G2 DNA damage checkpoint response, through the upregulation of the Bcl2 family proteins [20], or via induction of a quiescent state known as cancer dormancy, which may be an important mechanism for acquisition of drug resistance by cancer cells [21]. These dualistic effects of p38α also occur in skin carcinogenesis. Autophagy-associated decreased p38 phosphorylation enhances cell survival and UVB-induced SCC carcinogenesis in murine models [22]. In contrast, chronic UV irradiation of p38α-dominant negative (p38α DN) mice diminished the growth of skin tumors [23]. The cause of these disparate effects are not clear but interplay with other signaling pathways, as well as the nature of p38 MAPK substrates, could account for them [24]. For example, p38 activates the p53 tumor suppressor, and the p53 pathway is known to synergize p38 MAPK signaling, suggesting cross-talk between these two pathways [25]. Nonetheless, UV-induced mutational inactivation of p53 is a common finding in sun-exposed skin, and the majority of human SCCs harbor p53 mutations. Using p53-deficient SCC keratinocytes and p53^-/-^/SKH-1 mice, we assessed the role of p38α to explore the connection between p38α and NOX-mediated ROS generation. Our results indicate that chemical inhibition of p38 activity enhances UV-induced SCC growth in p53^-/-^/SKH-1 mice, which is accompanied by increased NOX2 expression and elevated intracellular ROS levels.

## Materials and Methods

### Cells and Reagents

A431 human epidermoid squamous cell carcinoma (SCC) cells were obtained from the American Type Culture Collection (ATCC, Manassas, VA) and maintained according to ATCC guidelines. Primary human keratinocytes isolated from neonatal foreskin were obtained from Columbia University Skin Disease Research Center (SDRC) tissue culture core facility and cultured in 154CF medium supplemented with human keratinocyte growth supplement (Life Technologies, Grand Island, NY). SB203580, a pyridinyl imidazole inhibitor widely used to inhibit the biological function of p38α/β [26]–[28], and diphenyleneiodonium (DPI), an inhibitor of NADPH oxidase-mediated ROS formation, were purchased from Sigma-Aldrich (St. Louis, MO). siRNA targeting p38α coding regions and scrambled siRNA controls (con) were obtained from Santa Cruz Biotechnology (Santa Cruz, CA). SB203580 was dissolved in DMSO for cell culture or in 0.5% methylcellulose (Sigma-Aldrich) for oral administration to mice. DPI was dissolved in dimethyl sulfoxide (DMSO) and added to cell cultures at a final concentration of 2.5 µM. SCC tissue microarrays (TMAs) were obtained from Imgenex (San Diego, CA).

### siRNA transfection

A431 cells were transfected with 40 nM of p38α MAPK siRNA or scrambled siRNAs using Lipofectamine RNAiMax (Santa Cruz Biotechnology, Santa Cruz, CA). The efficiency of p38α downregulation was assessed by RT-PCR and Western blotting 24 h–48 h after transfection.

### qRT-PCR and Western blotting

Total RNA was isolated from whole skin or cultured cells using the RNeasy Kit (Qiagen, Gaithersburg, MD) and treated with DNase I (Life Technologies, Grand Island, NY) according to the manufacturers' protocols. Total RNA (2 µg) was then reverse transcribed by Superscript III using random hexamer primers according to the manufacturer's instructions. Primers for each gene are listed in Table 1. For Western blotting, protein was isolated from whole skin or cultured cells following established procedures [29]. Proteins were resolved on 4–15% SDS-PAGE gels and blotted according to standard procedures using the following primary antibodies: p38α, p38β, p38γ, p38δ, p-p38 and cyclin D1 (Cell Signaling Technology, Danvers, MA), Cdc25C, and phospho-c-Jun (Santa Cruz Biotechnology, Dallas, TX), NOX2 (Abcam, Cambridge, MA), tubulin and β-actin (Sigma-Aldrich, St. Louis, MO).

**Table 1:** Primer sequences for real-time PCR.

	Forward (5′-3′)	Reverse (5′-3′)
GAPDH (human)	AATGAAGGGGTCATTGATGG	AAGGTGAAGGTCGGAGTCAA
p38α (human)	TCAGTCCATCATTCATGCGAAA	AACGTCCAACAGACCAATCAC
p38β (human)	AAGCACGAGAACGTCATCGG	TCACCAAGTACACTTCGCTGA
p38γ (human)	CATGAGAAGCTAGGCGAGGAC	CAGCGTGGATATACCTCAGCC
p38δ (human)	GCCGAGATGACTGGCTACG	TGGTCCAGGTAATCTTTCCCC
Nox2-1st (mouse)	ACCCTTTGGTACAGCCAGTG	TTGCAATGGTCTTGAACTCG
Nox2-2nd (mouse)	CCTTTGCCTCCATTCTCAAG	GTGCACAGCAAAGTGATTGG

### BrdU incorporation assay

A431 cell growth and proliferation following p38α siRNA-treatment or scramble siRNA control (scr)-treatment were analyzed by BrdU incorporation, following the manufacturer's instructions (BD Biosciences, San Jose, CA). For labeling, 24 h after transfection, BrdU was added directly to the cell culture to a final concentration of 100 µM, and cultures were incubated for another 24 h, at which point cells were harvested, fixed, permeabilized, treated with DNase I, and stained with a FITC-conjugated anti-BrdU antibody (BD Biosciences, San Jose, CA). Fluorescence intensity was measured using a fluorescence plate reader.

### Cell invasion assay

The invasiveness of p38α-depleted and control SCC cells was assessed using BD BioCoat Matrigel Invasion Chambers, following the manufacturer's instructions (BD Biosciences, San Jose, CA). Briefly, 1×10^5^ cells/well were plated onto the six-well plate and allowed to grow overnight at 37°C. Cells invading the matrigel were stained and counted.

### Measurement of intracellular ROS

Intracellular ROS was assessed using a cell-permeable fluorogenic probe, 2′,7′-dichlorofluorescein diacetate (CM-H2DCF-DA) dye (Life Technologies), which detects hydrogen peroxide production. Briefly, 48 h following transfection of the p38α siRNA or scrambled siRNA into A431 cells, CM-H2DCF-DA was added to the cells at a final concentration of 5 µM and incubated for 15 min at 37°C in the dark. CM-H2DCF-DA is oxidized by cytoplasmic ROS to a green fluorescent CM-DCF compound. After 2 washes with PBS, cells were detached by trypsin-EDTA and immediately analyzed by flow cytometry; 1×10^4^ cells were collected and analyzed for each sample.

### Measurement of NADPH oxidase activity

NADPH oxidase activity was measured in plasma membranes obtained from A431 cells or tumor tissues. Specimens were incubated in hypotonic buffer supplemented with protease inhibitor cocktails (Sigma-Aldrich, St. Louis, MO) for 30 min on ice. Following sonication, the homogenate was centrifuged at 1000×g for 15 min at 4°C. The supernatant was collected and centrifuged at 12,000×g for 1 h at 4°C. The pellet consisting of crude membranes was resuspended in 50 µl 1× PBS supplemented with 0.9 mM CaCl_2_ and 0.5 mM MgCl_2_. After adding 200 µl of solution containing 0.8 mM glucose, 2 mM luminol and 500 U/ml horseradish peroxidase, the reaction mixture was incubated for 1 min at 37°C. Following the addition of 10 ng/ml of phorbol myristate acetate (PMA), the RLU of chemiluminescence were recorded every 30 seconds for a total of 90 minutes at 37°C using a luminometer.

### Immunohistochemical (IHC) staining and quantification

Skin SCC tissue arrays (TMAs, Imgenex, IMH-323) were used in the immunohistochemical assessment of p38α. Sections were treated with Antigen Unmasking Solution (Vector Labs, Burlingame, CA) prior to incubation with primary antibodies and detection with DAB, as previously described [29] [30]. Images were obtained using an Axioplan2 microscope. The signal intensity and the extent of staining were quantified using the pixel analysis function of Adobe Photoshop. The resulting pixel values from each image were subjected to an unpaired t-test with Welch's correction by using GraphPad Prism software, to determine statistical significance.

### UV light source

A UV Irradiation Unit (Daavlin, Bryan, OH), equipped with an electronic controller to regulate the dosage, was used. The UV source consisted of eight FS72T12-UVB-HO lamps that emit UVB (290–320 nm, 75–80% of total energy) and UVA (320–380 nm, 20–25% of total energy). A UVC sensor (Goldilux UVC Probe, Oriel, Stratford, CT) was routinely used during each exposure to confirm lack of UVC emission. The UV dose was quantified with a UVB Spectrum 305 Dosimeter obtained from Daavlin. The radiation was further calibrated with an IL1700 Research Radiometer/Photometer from International Light (Newburyport, MA).

### Tumor study in p53^-/-^/SKH-1 mice

p53^-/-^/SKH-1 mice were generated as previously described [31] by crossing eight-week-old female non-agouti *p53* ^−/−^ mice (B6/C57BL/6J-Trp53^−/−^, Jackson Laboratories, Bar Harbor, ME) with SKH-1 males (Charles River Laboratories, Wilmington, MA) to generate littermates heterozygous for p53. These mice were then backcrossed for 11 generations to SKH-1 mice to minimize the C57BL/6 genetic background, and a colony of p53^−/−^/SKH-1 mice (F14–F16) were utilized for the tumorigenesis studies. Male or female 8–9 week-old p53^-/-^/SKH-1 mice were divided into four groups: Mice in Groups I and II received either 0.5% methylcellulose by gavage or UV irradiation (180 mJ/cm^2^), respectively. Mice in Group III received SB203580 (50 mg/kg body weight in 0.5% methylcellulose, gavage) and mice in Group IV received both UV irradiation and SB203580. SB203580 and/or UV irradiation were applied twice per week for a total of 22 weeks. Tumors were counted weekly once they reached 2 mm in diameter. At week 22, all mice were sacrificed, their dorsal skin removed, and tumors were harvested and collected for analysis.

### Xenografts of A431 cells

NOD/Shi-scid IL2rgamma(null) (NOG) mice were divided into four groups of six mice each. Groups I and II received 0.5% methylcellulose by gavage. Mice in Group III and IV received SB203580 by gavage (50 mg/kg body weight in 0.5% methylcellulose, three times per week for six weeks). Three days after starting the drug administration, A431 cells transduced with control vector (shCTRL) (3×10^4^ cells in 100 µl matrigel) were subcutaneously injected into mice in groups I and III, while mice in groups II and IV received NOX2 knockdown A431 cells (shNOX2). Tumor growth was measured every five days for 40 days.

### Ethics Statement

The tumor study in p53^-/-^/SKH-1 mice was carried out in strict accordance with the recommendations in the Guide for the Care and Use of Laboratory Animals of the National Institutes of Health. The protocol was approved by Columbia University Institutional Animal Care and Use. The xenograft study was performed at the University de Bordeaux; the protocol was approved by the animal ethics committee there. All efforts were made to minimize suffering in experimental animals.

### Statistics

Statistical analyses were performed using the Student's t test (two-tailed) or 1-way ANOVA tests, followed by post-hoc Tukey's tests. P<0.05 was considered significant. Results are presented as mean ± SD. Statistical analyses for TMAs were performed using an unpaired t-test with Welch's correction by using GraphPad Prism software, to determine the statistical significance of the difference between the SCC sample and the normal skin sample groups.

## Results

### Differential regulation of p38α MAPK expression in normal human keratinocytes (NHK) and A431 SCC cells following UV irradiation

**Figure 1:**
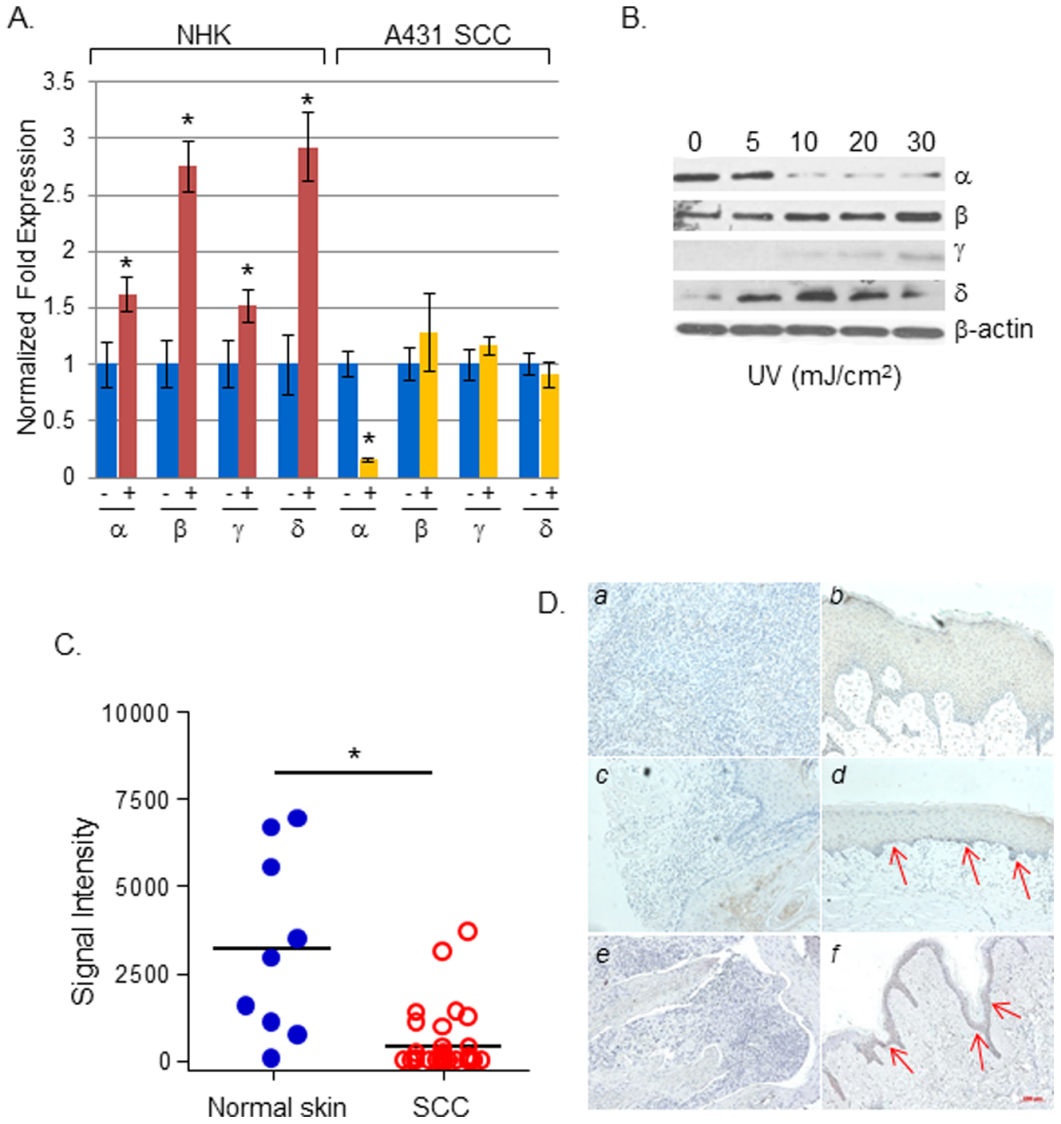
p38α MAPK expression is diminished in UV-irradiated A431 SCC keratinocytes and human SCCs. UV decreases p38α expression in A431 cells. The mRNA levels of p38α, β, γ, and δ were determined by real-time qRT-PCR in normal human keratinocytes (NHK) and human A431 cells, 24 h after UV irradiation (30 mJ/cm^2^).; The data shown are from a representative experiment of three different sets of independent experiments using keratinocytes derived from independent donors. *p<0.05. (B) The levels of p38 isoforms in A431 cells were assessed by Western blotting, 24 h after UV irradiation at the indicated UV doses. β-actin was used as an internal loading control; 50 µg protein was loaded per lane. (C) Immunohistochemical analyses of p38α in human cutaneous SCCs. p38α expression in cutaneous SCC tissue arrays containing 35 SCC sections and nine normal skin sections was analyzed by immunohistochemical staining. The signal intensity and the extent of staining were quantified using the pixel analysis function of Adobe Photoshop. Two SCC samples were excluded from the following statistical analysis, due to excessive infiltrations of p38α-positive lymphocytes in the tumor tissues. The resulting pixel values from each image were subjected to an unpaired t-test with Welch's correction by using GraphPad Prism software, to determine the statistical significance of the difference between the SCC sample and normal skin sample groups. *: p = 0.014. (D) Representative immunohistochemical staining of p38α. (*a*) Moderately differentiated and (*c*) well-differentiated cutaneous SCCs; (*b* and *d*) tumor-adjacent skin of (*a*) and (*c*), respectively; Representative immunohistochemical staining of phospho-p38. SCC (*e*) and its tumor-adjacent skin (f). Arrows indicate positive staining.

In previous studies, we showed that acute UV irradiation of the skin of SKH-1 mice activates p38 MAPK signaling, which transiently increases the local pro-inflammatory response [32]. p38α was recently shown to be the dominantly phosphorylated p38 isoform in response to UV in hTERT-immortalized human keratinocytes [23], suggesting a role in UV-induced skin carcinogenesis. We first assessed the effect of UV irradiation on p38 isoforms in NHK and A431 SCC cells. In Fig. 1A, we show that acute UV irradiation of NHK increased the expression of all four p38 isoforms (α/β/γ/δ), but particularly p38β and p38δ. In A431 cells, p38α showed a selective reduction in response to UV (Figs. 1A, 1B), whereas p38β showed a slight increase, as assessed by Western blotting (Fig. 1B). The level of p38δ substantially increased in UV-irradiated A431 cells compared to non-irradiated controls, while the levels of p38γ were relatively low in both the non-irradiated and UV-irradiated A431 cells. Additionally, while the siRNA-mediated knockdown of p38α had no significant effects on other p38 isoforms in NHK, p38α knockdown in A431 cells led to the upregulation of p38β and p38δ, as determined by real-time PCR (Fig. S1). These data suggest that the decrease in p38α causes compensatory increases in p38β and p38δ in A431 cells. Taken together, our results indicate that p38 isoforms are differentially regulated in response to UV irradiation, in which p38α is selectively downregulated in A431 cells.

### p38α is downregulated in human SCCs

The immunohistochemical assessment of human cutaneous SCC tissue microarrays demonstrated that the majority of SCCs either lacked or had reduced p38α expression (Fig. 1C, normal vs. SCCs, *p* = 0.014). Representative pictures of p38α immunohistochemical staining in paired tumor and tumor-adjacent skin (Fig. 1D) indicate that p38α is present in both the basal and suprabasal layers of non-tumor bearing epidermis (*b, d*), whereas it was substantially reduced in SCCs (*a, c*). Furthermore, phosphorylated p38 levels were substantially diminished in SCCs (e, *f*).

### Pharmacological inhibition of p38α MAPK enhances proliferation of A431 SCC cells lacking functional p53

**Figure 2:**
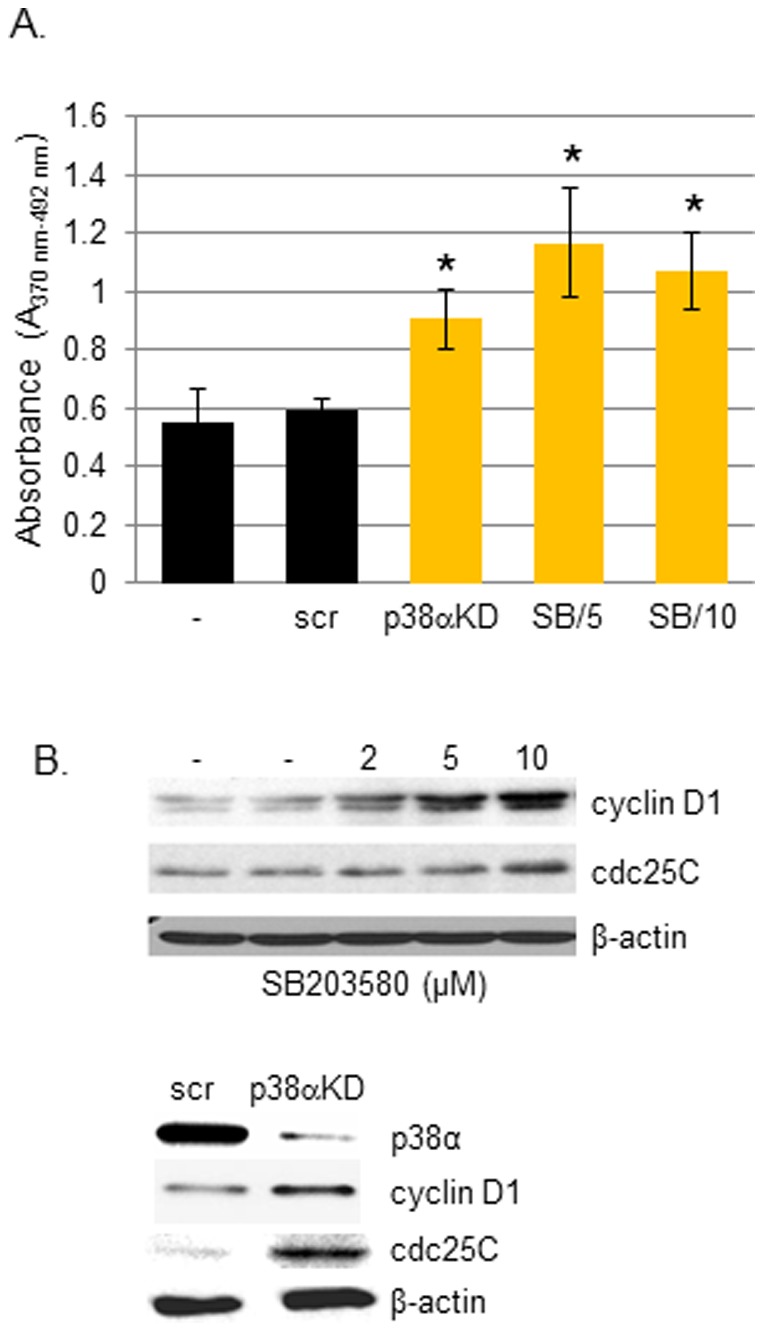
p38α inhibition enhances proliferative capacity of A431 SCC keratinocytes. (A) The effects of p38α inhibition were assessed by BrdU incorporation in A431 cells transfected with p38α siRNA (p38αKD), or treated with SB203580 (5 µM (SB/5), 10 µM (SB/10) for 24 h. Each histogram represents the results from triplicate cultures; *p<0.05. scr, scrambled control siRNA. The levels of cyclin D1 and cdc25C in A431 cells treated with the indicated concentrations of SB203580 for 24 h (B), or following p38α knockdown (C) were assessed by Western blotting (50 µg protein loaded per lane). β-actin was used as an internal loading control.

p38α influences UV stress responses by several pathways, including its effects on p53. It is known that p53 mutations occur early during the induction of UV-induced SCCs in humans as well as in murine models [33], [34]. To determine the effects of p38α deficiency in the absence of p53, we used p38α-expressing A431 cells harboring mutant p53. Both genetic inhibition of p38α via siRNA-mediated p38α knockdown and chemical inhibition of p38 activity using SB203580 resulted in significant increases in the proliferation of A431 cells, as measured by BrdU incorporation (Fig. 2A). This was associated with increased cyclin D1 and cdc25C (Fig. 2B), which are cell cycle regulators known to be upregulated in SCC carcinogenesis [30]. These results suggest that inhibition of the p38 signaling pathway, which likely involves p38α, drives cell proliferation.

### Inhibition of NADPH oxidase decreases proliferation and invasiveness of p38α-deficient A431 SCC cells

**Figure 3:**
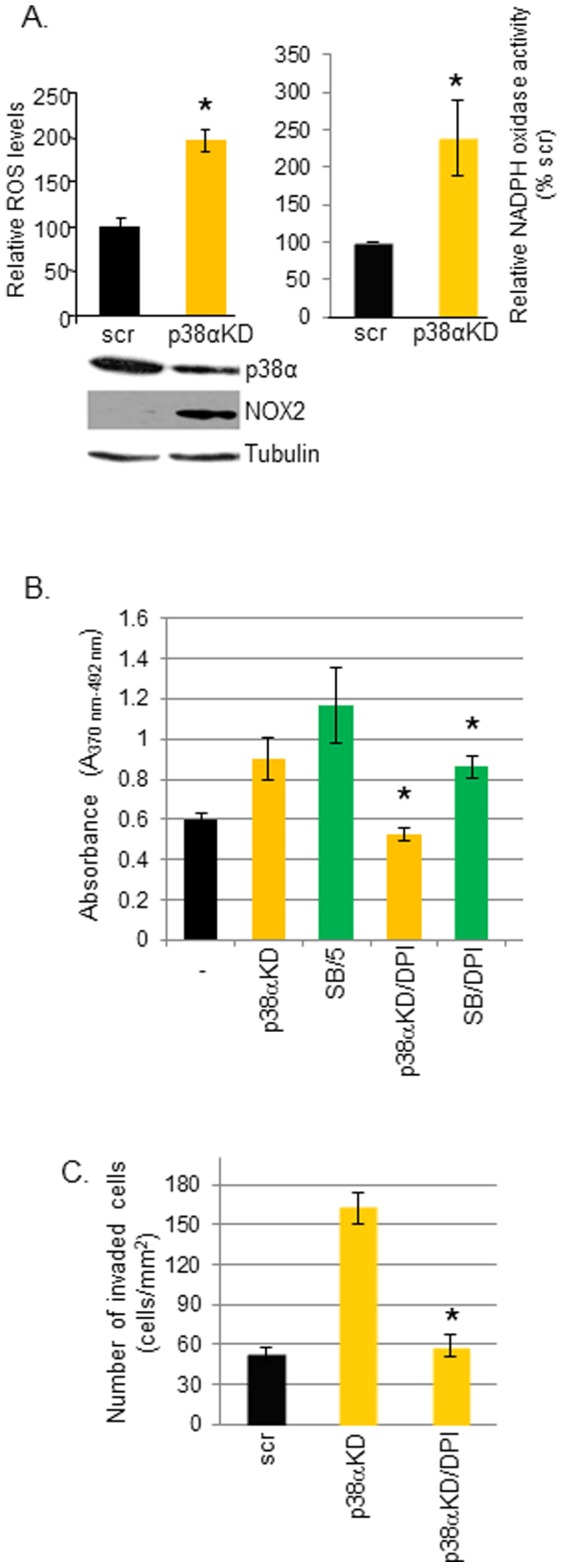
Inhibition of NADPH oxidase activity reduces the cellular proliferation and invasiveness of p38α-deficient A431 SCC keratinocytes. (A) p38α downregulation increases NADPH oxidase activity and generates intracellular ROS. Intracellular ROS levels were measured using a cell-permeable fluorogenic probe, 2′,7′-dichlorofluorescein diacetate (DCFDA) dye, which detects hydrogen peroxide production, following siRNA-mediated p38α knockdown in A431 cells. NOX2 levels were assessed by Western blotting in extracts prepared from p38α knockdown (p38αKD) A431 cells. NADPH oxidase activity was determined as previously described [11]. scr, scrambled control siRNA. Presence of NADPH oxidase inhibitor, DPI (2.5 µM), inhibits the proliferation (B) and cellular invasiveness (C) of A431 cells treated with SB203580 or p38αKD A431 cells. Proliferation was measured by a BrdU incorporation assay, and invasiveness was assessed in matrigel-coated chambers. Each histogram represents results from triplicate cultures; *p<0.05.

We previously showed that NOX1 is overexpressed in human SCCs [10]. In A431 cells, p38α knockdown increased NOX2 expression and NADPH oxidase activity that was associated with increased ROS production (Fig. 3A). Pretreatment of A431 cells with DPI, a NADPH oxidase inhibitor, abolished the increased cell proliferation and invasiveness observed in p38α knockdown and SB203580-treated A431 cells (Fig 3B, C) suggesting a link between NOX2-dependent ROS generation and cell proliferation and *in vitro* invasiveness.

### Oral administration of the p38 MAPK inhibitor SB203580 enhances UV-induced skin carcinogenesis in p53^-/-^/SKH-1 mice

**Figure 4:**
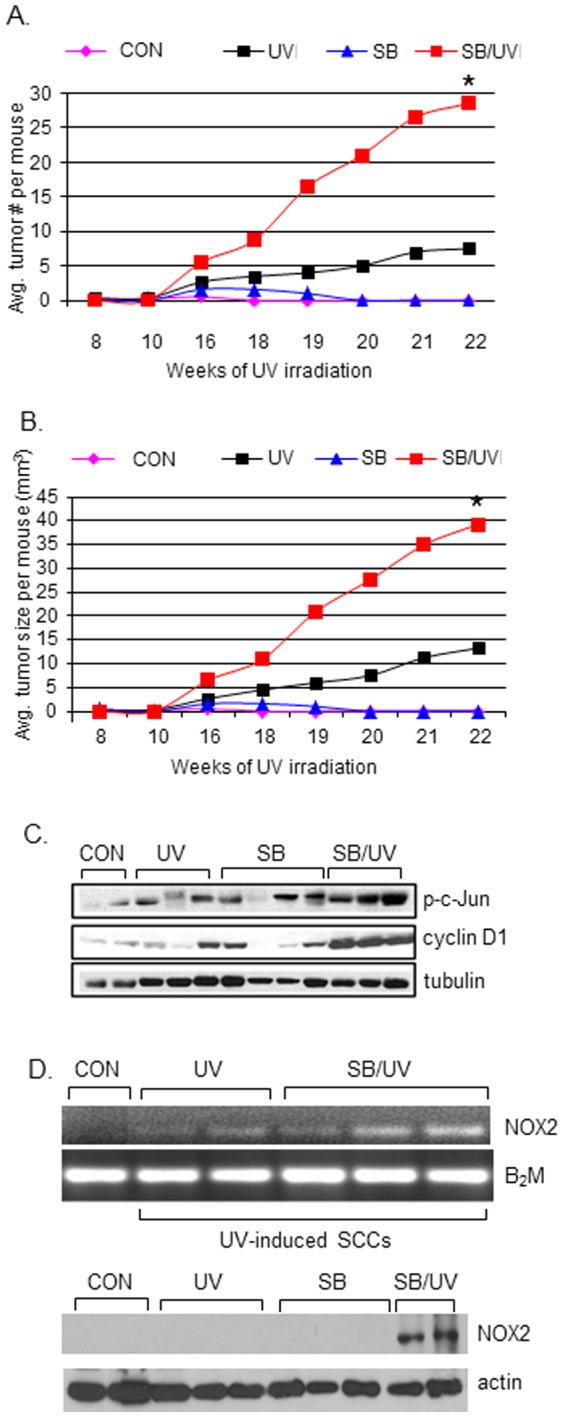
The p38 MAPK inhibitor SB203580 accelerates UV-induced skin carcinogenesis and increases NOX2 levels in p53^-/-^/SKH-1 mice. The number of skin tumors (A) and tumor size (B) at week 22 in UV-irradiated p53^-/-^/SKH-1 mice (UV) were compared to mice that had received SB203580 alone (SB) or received both SB203580 and UV irradiation (SB/UV). control, non-irradiated, non-treated mice; *p<0.05, UV vs. SB/UV. (C) The levels of phospho-c-Jun and cyclin D1 in tissue extracts, assessed by Western blotting. 60 µg total protein per lane; β-actin was used as an internal loading control. (D) SB203580 administration leads to the upregulation of NOX2 in UV-induced SCCs in p53^-/-^/SKH-1 mice. NOX2 levels were detected by RT-PCR (top panel) and Western blotting (bottom panel). B_2_M and actin were used as internal controls for RT-PCR and Western blotting, respectively.

To elucidate the role of p38 in the pathogenesis of SCCs in the absence of p53, we utilized our p53^-/-^/SKH-1 mouse model in a standard photocarcinogenesis protocol. SB203580 was administered orally (50 mg/kg) prior to each UV irradiation (180 mJ/cm^2^, twice per week for a total of 22 weeks). As compared to UV-irradiated mice, SB203580-treated UV-irradiated mice showed a four-fold increase in the number of skin tumors (Fig. 4A) and a three-fold increase in average tumor size (Fig. 4B). No tumors developed in unirradiated SB203580-treated mice. Interestingly, liver-specific deletion of p38α is known to enhance JNK activity and the levels of c-Jun, leading to hepatocyte proliferation and hepatic tumorigenesis [35]. Similarly, we found elevated phosphorylated c-Jun and cyclin D1 in SCCs harvested from the UV-irradiated, SB203580-treated mice (Fig. 4C). Importantly, SB203580 administration augmented the mRNA and protein levels of NOX2 (Fig. 4D) and NADPH oxidase activity (Fig. 4E), suggesting that inhibition of p38 may exacerbate the carcinogenic effects of UV by driving NOX2-dependent ROS signaling.

### NOX2 downregulation suppresses the growth of SB203580-treated A431 xenograft tumors

**Figure 5:**
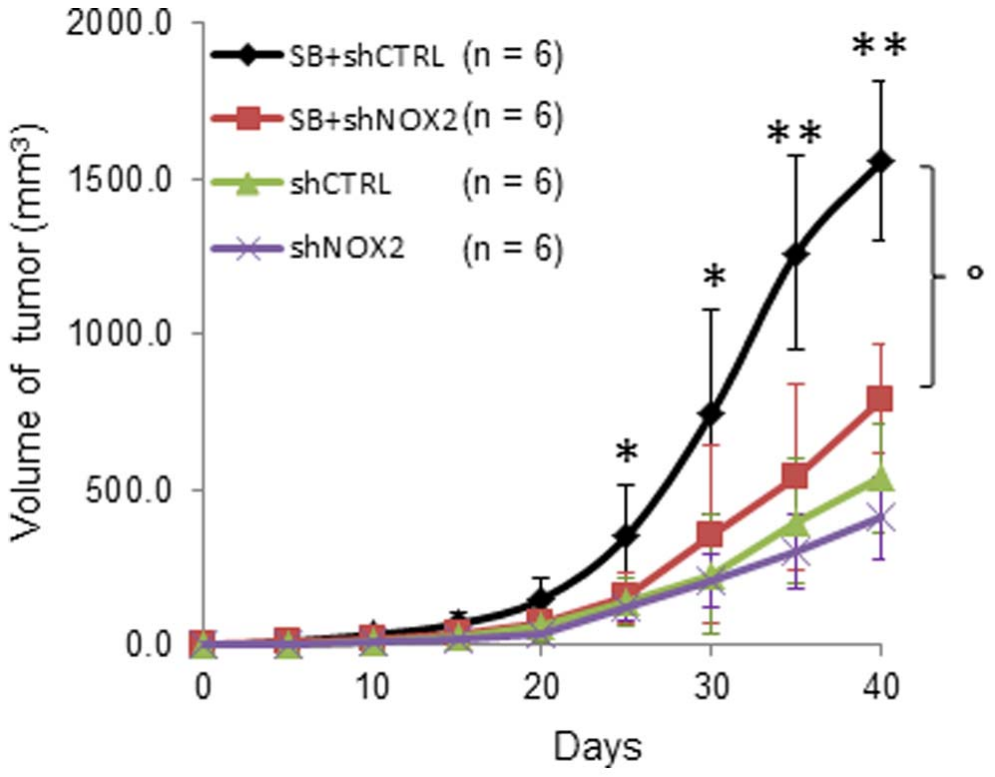
NOX2 silencing suppresses the growth of A431 xenografts treated with SB203580. The *in vivo* tumor growth of NOX2 knockdown A431 cells subcutaneously injected into NOG mice, which were either treated or not treated with SB203580 (SB+shNOX2 or shNOX2, respectively). A431 cells were transduced three days prior to injection. shCTRL, A431 cells transduced with control shRNA; SB+shCTRL, A431 cells transduced with control shRNA and treated with SB203580. Results are presented as mean ± SD. * *p*<0.05, **p<0.001 SB+shCtrl vs. shCtrl; ° *p* = 0.032, SB+shCtrl vs. SB+shNOX2.

To determine whether NOX2 influences tumor growth, A431 cells transduced with shRNA targeting NOX2 (shNOX2) or control shRNA (shCTRL) were subcutaneously injected into immunodeficient NOG mice (n = 12 each group). These mice were further divided into two groups of six mice each, of which one group received SB203580 (50 mg/kg, gavage, three times per week for six weeks), while the other groups received a methylcellulose vehicle. Consistent with the data observed in UV-irradiated p53^-/-^/SKH-1 mice in Fig. 4, the growth of A431 xenografts were substantially enhanced in mice treated with SB203580 (Fig. 5; shCTRL vs. SB+shCTRL). NOX2 silencing suppressed this growth by 50% (SB+shCTRL vs. SB+shNOX2). Interestingly, NOX2 silencing alone did not significantly affect the growth of A431 xenografts (shNOX2; Fig. 5). These data indicate a direct relationship between p38 and NOX2 and suggest NOX2 as a potential therapeutic target for SCCs with diminished p38 activity.

## Discussion

p38α is generally considered to be a tumor suppressor; however, studies performed in various model systems suggest that p38α may have dualistic or context-dependent effects in regulating cell death and survival, in part due to substrate specificity and sensitivity in response to specific stimuli. Because UV-induced mutational inactivation of p53 is an important driver of cutaneous SCCs, we employed p53-deficient A431 SCC cells and a p53^-/-^/SKH-1 mouse model to assess the p53-independent role of p38α in skin carcinogenesis. Genetic inhibition of p38α or pharmacological inhibition of p38 activity with SB203580 increased the proliferation and invasiveness of A431 cells (Figs. 2, 3), and the oral administration of SB203580 augmented the growth of UV-induced SCCs in p53^-/-^/SKH-1 mice (Fig. 4). Further confirmation of our results comes from our observation that p38α is decreased in human primary SCCs compared to non-tumor bearing skin and in UV-irradiated A431 cells (Fig. 1), suggesting its role in tumor suppression. Our results are consistent with those of Qiang et al [22], who showed that autophagy-associated decreases in p38 phosphorylation enhances cell survival [22]. p38 activity was also found to be reduced in human SCCs, compared to normal human skin [22]. Moreover, inhibition of p38 signaling was associated with a defect in global genome nucleotide excision repair (GG-NER), a vitally important tumor protective pathway that recognizes and excises UVB-induced CPDs (cyclobutane pyrimidine dimers) and 6-4PPs (pyrimidine (6-4) pyrimidone photoproducts) that would otherwise be mutagenic [36]. These data indicate that p38α loss can promote UV-induced skin tumorigenesis. Our results, however, differ from those of Dickinson et al. who reported that a dominant negative mutant of p38α (p38αDN) SKH-1 mice, consisting of T180A and Y182F point mutations at the Thr-Gly-Tyr activation site [26], [37], were resistant to UV-induced skin carcinogenesis [37]. Similarly, using the same p38αDN model, Dong et al demonstrated that p38 blockade resulted in fewer and smaller tumors in response to ultraviolet radiation [38]. The explanation for these conflicting results is unclear, but several possibilities can be considered. For example, our study utilized p53^–/–^/SKH mice and cultured p53-mutant human cells. Given the synergistic interaction between p38 and p53 in cell-cycle regulation [25], we postulate that, in the absence of p53, p38α could compensate for the loss of the p53 tumor-suppression function; this may explain the augmented UV-induced skin carcinogenesis observed in SB203580-treated p53^–/–^/SKH mice. In addition to the p53 functional status, other factors could contribute to the observed discrepancy: differential modes of p38 inhibition, the role of other p38 isoforms, differential inflammatory responses, and the source of UV (i.e., the UV radiation employed in our study comprised 75–80% UVB and 20–25% UVA of total energy, whereas Dong and colleagues used 95% UVA and 5% UVB [38]).

While p38α has been shown to be predominantly sensitive to SB203580 in certain cases—for example, in primary fibroblast [27]—SB203580 is known to target both p38α and p38β isoforms. Coupled with our data showing the presence of p38β and its UV-induced increase in A431 cells (Fig. 1), as well as a compensatory increase of p38β following p38α knockdown (Fig. S1), it is possible that the effect of SB203580 can be attributed to the inhibition of both p38α and p38β, and not solely p38α. Additionally, it is interesting to note that the p38δ level increased in p38α knockdown A431 cells; no such effects were seen in normal human keratinocytes (Fig. S1). The p38δ level was also increased in UV-irradiated A431 cells (Fig. 1B). In a study that employed two-stage 7,12-dimethylbenz(a)anthracene/12-O-tetradecanoylphorbol-13-acetate chemical skin carcinogenesis, mice lacking p38δ were resistant to the development of benign papillomas [39]; this suggests that p38δ promotes tumorigenesis. Whether p38δ indeed promotes SCC tumorigenesis and whether it plays a similar role in UV-induced skin carcinogenesis remains unknown. Nonetheless, these data collectively suggest a complex interplay among p38 isoforms during skin responses to UV radiation and warrant further investigation, if we are to understand the functional relevance of these compensatory increases in UV carcinogenesis and the specific contribution of the individual p38 isoforms that underlie skin cancer pathogenesis.

Immunohistochemical assessments of p38α in human cutaneous SCC tissue arrays indicate that p38α levels are reduced in tumors, compared to non-tumor-bearing skin (Fig. 1). The mechanism underlying p38α downregulation is not clear. Recently, it was shown that UV irradiation of HaCaT cells, a spontaneously immortalized skin keratinocyte cell line, harboring mutant p53 induces NF-κB-mediated miR-125b expression, which repressed p38α levels by targeting its 3′-UTR [17]. miR-125b-mediated p38α repression was also shown to protect cells against UV-induced apoptosis, thereby promoting cell survival [17]. The combination of the absence of functional p53 and UV-induced p38α repression likely provide a survival advantage that accelerates tumor promotion and progression. Further research assessing the status of p38α and p53 in tumors developed in p38αDN mice and p53^-/-^/SKH-1 and p53^+/+^/SKH-1 mice in response to UV may help clarify the relationship between p38α and p53 in skin carcinogenesis.

It is important to note, however, that a previous study using high-density oligonucleotide arrays identified p38α expression as being moderately increased (1.4-fold) at the mRNA level in human cutaneous SCCs [40]. Junttila and colleagues also reported that p38α mRNA is expressed in head and neck SCC (HNSCC) cell lines at levels comparable to that in normal squamous epithelial cells [15]. Since our results were based on IHC—to facilitate comparisons of protein levels between cutaneous SCCs and normal skin—the lack of p38α protein in our study could indicate a possible post-transcriptional control of p38α during SCC tumorigenesis.

Our results indicate that p38α loss during SCC pathogenesis is accompanied by enhanced NOX2 expression leading to increased intracellular ROS levels and that NOX2 downregulation suppresses the growth of A431 xenografts (Figs. 3, 5). Given that the p38α protein does not possess DNA-binding activity, its role in NOX2 regulation is likely to be indirect and perhaps involves transcription factors known to be direct targets of p38 [41]. Additionally, p38α phosphorylates serine residues in the N-terminal tail of histone H3 (Ser10 and Ser28) [42] [43], suggesting a potential for epigenetic reversible regulation of gene expression [44] [45]. In A431 cells, p38α knockdown increased the DNA-binding activity of the transcription factor AP-1, and we have identified putative AP-1 recognition sites in the NOX2 promoter (data not shown). Further studies are needed to define the mechanisms by which p38α regulates NOX2 expression and the relevance to UV-induced skin tumorigenesis. Identification of UV-specific p38α substrates, as well as modulation of p38α expression in genetically modified murine skin cancer models in a time- and tissue-specific manner, will aid in understanding its effects on signaling pathways that are relevant to skin carcinogenesis and may also help to determine whether restoration of p38α expression can prevent the development of these tumors.

## Supporting Information

Figure S1 **siRNA-mediated knockdown of p38α has no significant effects on other p38 isoforms in NHKs (left panel), but led to compensatory upregulation of p38β and p38δ in SCC cells (right panel).**
(TIF)
