# Correction: Inhibition of p38 MAPK Signaling Augments Skin Tumorigenesis via NOX2 Driven ROS Generation

**DOI:** 10.1371/journal.pone.0172451

**Published:** 2017-02-14

**Authors:** Liang Liu, Hamid Reza Rezvani, Jung Ho Back, Mohsen Hosseini, Xiuwei Tang, Yucui Zhu, Walid Mahfouf, Houssam Raad, Grace Ragi, Mohammad Athar, Arianna L. Kim, David R. Bickers

The ninth author's name is spelled incorrectly. The correct name is: Grace Ragi.
