# Supplementary figures and images for: Inhibition of p38 MAPK Signaling Augments Skin Tumorigenesis via NOX2 Driven ROS Generation
Source: PLoS One. 2014 May 13;9(5):e97245. doi: 10.1371/journal.pone.0097245 (PMC4019556; doi:10.1371/journal.pone.0097245)

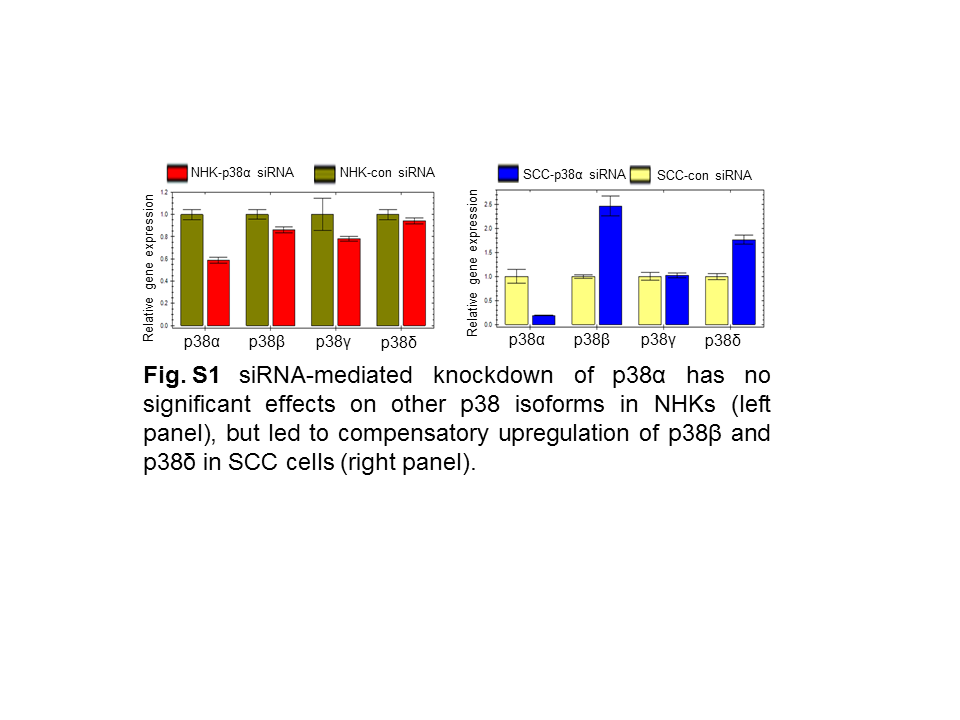

Supplement: Figure S1 — siRNA-mediated knockdown of p38α has no significant effects on other p38 isoforms in NHKs (left panel), but led to compensatory upregulation of p38β and p38δ in SCC cells (right panel). (TIF) [file pone.0097245.s001.tif]
